# DEAD-Box Helicase DDX25 Is a Negative Regulator of Type I Interferon Pathway and Facilitates RNA Virus Infection

**DOI:** 10.3389/fcimb.2017.00356

**Published:** 2017-08-04

**Authors:** Tingting Feng, Ta Sun, Guanghao Li, Wen Pan, Kezhen Wang, Jianfeng Dai

**Affiliations:** Jiangsu Key Laboratory of Infection and Immunity, Institute of Biology and Medical Sciences, Soochow University Suzhou, China

**Keywords:** DDX25, interferon, innate immune response, dengue virus, IRF3, NFκB

## Abstract

Dengue is a mosquito-borne viral disease that rapidly spread in tropic and subtropic area in recent years. DEAD (Glu-Asp-Ala-Glu)-box RNA helicases have been reported to play important roles in viral infection, either as cytosolic sensors of viral nucleic acids or as essential host factors for the replication of different viruses. In this study, we reported that DDX25, a DEAD-box RNA helicase, plays a proviral role in DENV infection. The expression levels of DDX25 mRNA and protein were upregulated in DENV infected cells. During DENV infection, the intracellular viral loads were significantly lower in *DDX25* silenced cells and higher in *DDX25* overexpressed cells. Meanwhile, the expression level of type I interferon (IFN) was increased in *DDX25* siRNA treated cells during viral infection. Consistent with the *in vitro* findings, the *Ddx25*-transgenic mice have an increased susceptibility to lethal vesicular stomatitis virus (VSV) virus challenge. The viremia was significantly higher while the anti-viral cytokine levels were lower in *Ddx25*-transgenic mice. Further, DDX25 modulated RIG-I signaling pathway and blocked IFNβ production, by interrupting IFN regulatory factor 3 (IRF3) and NFκB activation. Thus, DDX25 is a novel negative regulator of IFN pathway and facilitates RNA virus infection.

## Introduction

Dengue virus (DENV) is a mosquito-borne viral pathogen, which is mainly transmitted by *Aedes aegypti* and *Aedes albopicuts*. Dengue imposes a significant burden on human health around the world and is endemic in more than 100 countries in tropical and subtropical areas, especially in Southeast Asia, the Americas, the Western Pacific, Africa, and Eastern Mediterranean regions (Guzman and Harris, 2015). Currently, there is no effective treatment for DENV infections. The first DENV vaccine was recently licensed for use after several decades of efforts, unfortunately, it confers only partial protection to all DENV serotypes (Gan, 2014; Thisyakorn and Thisyakorn, 2014).

Viruses have limited genetic capacity and as such rely on cellular factors to complete their life cycle. Thus, viruses interact with cellular proteins to acquire activities not encoded in the viral genome, to evade host immune defenses, and to manipulate cellular pathways to facilitate their replication (Khadka et al., 2011). However, while viruses attempt to hijack host cell machinery, the host cells attempt to halt, or slow down viral efficacy. DENV infection would normally activate the host antiviral response, in which the type I interferon pathway is crucial for host natural immunity. RNA helicases of DEAD-box protein family modulate the RNA structure and are crucial in many fundamental biological processes (de la Cruz et al., 1999; Rocak and Linder, 2004; Jankowsky, 2010). In addition to the traditional functions in RNA metabolism, DEAD-box RNA helicases have been reported as mediators of anti-viral innate immunity, or essential host factors for viral replication (Steimer and Klostermeier, 2012; Fullam and Schroder, 2013). Thus, DEAD-box RNA helicases family members play either a proviral or antiviral role during viral infection.

DDX3 is one of the first host cell DEAD-box RNA helicases identified as flavivirus cofactor, which is important for replication of viral RNA (Ariumi et al., 2007). For example, DDX3 colocalizes with NS3 near the nucleus during West Nile virus infection (Chahar et al., 2013). Another cellular helicase, DDX1 can directly bind to poly (I:C) (Zhang et al., 2011a). DDX21 and DHX36 are located downstream of DDX1 and both DDX21 and DHX36 interact with the downstream protein TIR-domain-containing-adapter-inducing interferon-β (TRIF). This suggests that DDX1 senses dsRNA and then triggers signaling via DDX21 and DHX36 to TRIF (Zhang et al., 2011a). DDX1 has also been reported to be important for the human immunodeficiency virus type 1 (HIV-1) replication as it binds to and serves as a cofactor of the HIV-1 Rev protein (Fang et al., 2004). DDX41 binds to STING (stimulator of interferon genes), which are located on the endoplasmic reticulum membrane (Zhang et al., 2011b; Parvatiyar et al., 2012).

In our previous study, 40 genes of DEAD-box family were screened using the RNAi approach to identify the putative host factors for DENV infection. Our result showed host DDX family members, DDX3X and DDX25, played an antiviral and a proviral role respectively during DENV infection (Li G. et al., 2015). DDX25, also named as Gonadotropin-regulated testicular RNA helicase (GRTH), is well-known as an essential testis-specific protein for completion of spermatogenesis in mouse model (Kavarthapu and Dufau, 2015). The expression profile and the role of DDX25 during viral infection has not been reported yet. We here reported that DDX25 interrupts the IFN-signaling pathway by inhibiting IRF3 and NFκB activation, which negatively regulated IFNβ activity, thereby facilitates RNA virus infection.

## Materials and methods

### Ethics statements and mice

C57BL/6 adult wild type mice (ages 6–8 week) were purchased from Shanghai Laboratory Animal Center (Shanghai, China). C57BL/6-*Ddx25* transgenic mice (*Ddx25-*Tg) were produced by Shanghai Biomodel Organism Science & Technology Development Co., Ltd. (Shanghai, China). Microinjections and mouse transgenesis experiments were performed by inserting CAG-*Ddx25*-IRES-Luciferase-polyA plasmid into the genome of fertilized egg of C57BL/6 mice using a PiggyBAC Transposon system. Mice were genotyped by tails snips and PCR at 3 weeks of age. The genotyping primers were TCCAAGGGGCACCGAAGTCACCAA(forward), and GCGCCGGGCCTTTCTTTATGTTTT(reverse).

All mice were housed in specific pathogen free facilities in accordance with the Guide of National Animal Care and Use committee. All animal experiments were approved by the Institutional Laboratory Animal Care and Use Committee of Soochow University.

### Virus, tissues, cell culture, and infection

DENV-2 virus (DENV New Guinea C stain) were propagated in mosquito C6/36 cells (ATCC® CRL-1660). Sendai virus (SeV) was propagated in 10 days old embryonated chicken eggs (Bejing Laboratory Animal Research Center, Beijing, China), and the virus titer was detected by hemagglutination assay using chicken red blood cells (BeNa Culture Collection, Bejing, China). The VSV and VSV-green fluorescent protein (GFP) virus that expresses GFP as a non-structural protein was provided by Dr. Chunsheng Dong (Soochow University). The VSV-GFP virus was grown on a Vero E6 cell in Dulbecco's modified Eagle's medium (Invitrogen, Carlsbad, CA) containing 2% fetal bovine serum, titrated in Vero E6 cells.

The HEK293T cells were cultured in Dulbecco's modified eagle medium supplemented with fetal bovine serum (10%) and penicillin/streptomycin (1%). Cells were maintained at 37°C in a 5% CO_2_ laboratory incubator that was routinely cleaned and decontaminated. HEK293T cells were infected with DENV, SeV, or VSV-GFP at a multiplicity of infection (MOI) of 1, unless otherwise stated.

For *in vivo* VSV intranasal infections, 1 × 10^6^ *pfu* of VSV in 50 μl of endotoxin-free PBS were inhaled by isoflurane-anesthetized C57BL/6 wild type and *Ddx25-*Tg mice, with PBS-only as control. Thereafter, mice were monitored daily for weight loss and symptoms of disease.

Human tumor tissue samples were obtained from the First Affiliated Hospital of Soochow University (Suzhou, China) under institutional guidelines and used in gene expression analysis.

### Plasmid constructs

Recombinant plasmid for DDX25 expression was constructed using standard protocols by inserting the *DDX25* open reading frame into the pcDNA3.1 vector. Reporter plasmids NFκB-luc and pRL-TK were purchased from Clontech (USA) and used for dual luciferase reporter assays. Luciferase reporter plasmids IFNβ-luc, IRF3-luc, and ISRE-luc were kindly provided by Dr. Rongtuan Lin, McGill University, Canada (Zhao et al., 2007).

### siRNA and transfections

Transfections of HEK293T cells with plasmid DNA and siRNA were conducted using Lipofectamine 2000 (Invitrogen, USA) according to the manual of the manufacturer. The siRNA sequences for human *DDX25* gene were target I-GCAGCTAATTCACTCTTAA, target II-GCAATGTTAAGCAGAGTTA, and target III-GCCACCAGGTGTCTTTGTT, respectively (RiboBio Co., Guangzhou, China). RNAi efficiency was confirmed through quantitative reverse transcription polymerase chain reaction (qRT-PCR). Then, the cells were infected with DENV at an MOI of 1 for 48 h (except for the cases noted in the text) to test the influence of DDX25 silencing on DENV replication.

### TCID_50_ assay and viral growth kinetics

The cell-free supernatants were collected and the titers of DENV and VSV were assayed with a TCID_50_ assay according to standard protocols on Vero cells (ATCC® CCL-81) as described previously (Wang et al., 2016). The viral replication levels inside cells, in terms of the transcript levels of the DENV-2 envelop gene (E) or VSV glycoprotein gene (G), were quantified by qRT-PCR and normalized to β*-actin* gene.

### RNA isolation and real-time PCR

Total RNA was isolated using an RNA extraction Kit (Omega, Netherlands) and reverse-transcribed into cDNA using the first strand cDNA Synthesis Kit (Takara, Japan). qRT-PCR was performed using a SYBR Green with gene-specific primers (Applied Biosystems, USA) and normalized to β*-actin* gene. (oligo-primer sequences for qRT-PCR of this study were shown in Table S1 in the Supplemental Materials).

### Luciferase reporter assays

For luciferase reporter assays, 70% confluent HEK293T cells were transfected with 10 ng of pRL-TK reporter plasmid (herpes simplex virus thymidine kinase promoter driving *Renilla* luciferase, internal control), 100 ng of IFNβ luciferase reporter plasmid (firefly luciferase, experimental reporter), 50 ng of IFNβ activators (RIG-IN, MAVS, TBK1, IKKε, IKKα, NFκB, or IRF3), as well as either 100 ng of recombinant expressing plasmids or siRNAs [Vector, DDX25, 50 nM negative control (N.C.), or DDX25 siRNA]. For measuring the activation of transcription factor NFκB and IRF3, NFκB and IRF3 responsive element specific reporter plasmids were used in the luciferase reporter assays. Subconfluent HEK293T cells were transfected with 10 ng of pRL-TK reporter, 100 ng of NFκB (pNFκB-Luc), or IRF3 (pPRD(III–I)-Luc) luciferase reporter plasmid, various doses of recombinant expression plasmids (Vector or DDX25), along with 50 ng of expression plasmids of RIG-IN. At 24 h post-transfection, cells were infected with DENV-2 at an MOI of 1 and incubated further for 24 h. Luciferase activity was measured using a Promega Dual Glow Kit according to the instructions of the manufacturer (Promega, USA).

### Immunofluorescence microscopy

HEK293T cells were transfected with siRNA (N.C. or DDX25 siRNA) using Lipofectamine 2000 to examine the effect of DDX25 silencing on DENV infection. At 24 h post-transfection, cells were infected with DENV-2 at an MOI of 1. Cells were fixed in 1% paraformaldehyde and permeabilized with 1% Triton X100 at 48 h post-infection. DENV envelope proteins were probed with mouse anti-DENV E antibody (Santa Cruz, USA) and stained with FITC-labeled anti-mouse IgG (Jackson ImmunoResearch). Cell nuclei were stained with DAPI. Cells were then examined using a fluorescence microscope.

### Western blot

HEK293T Cells were transfected with pcDNA-DDX25 (or pcDNA3.1 vector) and infected with DENV-2 at an MOI of 10. At 24 h post-infection, cell lysates were subjected to SDS-PAGE and transferred onto a PVDF membrane for western blotting. Non-reducing native PAGE was performed to detect the dimerization of IRF3. Briefly, cell lysates were prepared in a native sample buffer without SDS and electrophoresed on a 10% non-reducing polyacrylamide gel without SDS.

The following primary antibodies were used for western blotting: anti-human Actin polyclonal Ab (Proteintech, USA), His-tag polyclone antibody (GenScript, USA), IRF3 polyclonal antibody (Biolegend, USA), anti-IRF3 (phospho S386) Ab (Cell Signaling, USA). HRP-conjugated donkey anti-rabbit IgG and rabbit anti mouse IgG mAb (Biolegend, USA) were used as secondary antibodies. The signals were detected using an ECL detection system (Merck Millipore Ltd., USA).

### Enzyme-linked immunosorbent assay (ELISA)

The protein concentrations of IFNβ and TNFα in VSV infected mouse serum samples were measured by ELISA Kits according to the instructions of the manufacturer (R&D Systems).

### Histopathology

Two days post VSV infection, wild type and *Ddx25*-Tg mice were sacrificed and the lung tissues were fixed in 10% formalin and paraffin embedded. Hematoxylin and eosin (H&E) staining was conducted for histopathology.

### Statistical analysis

Statistical significances were calculated with an unpaired two tailed Student's *t*-test and Log-rank (Mantel-Cox) Test (for survival data only) using Prism 5 software (GraphPad).

## Results

### DDX25 is upregulated upon DENV infection

In mice, *Ddx25* was reported to be highly expressed in testis and critical for the process of spermatogenesis (Sheng et al., 2003). Contrastingly, by searching the gene expression database, we have found that human *DDX25* mRNA was expressed in almost all the human tissues (data from database of GTEx, BioGPS, Illumina Human BodyMap, and SAGE). Human DDX25 proteins are also detected in multiple tissues, such as testis, spleen, tonsil, ovary, and retina, as well as various cell lines including A549, HEK293T, SNB-75, JUNKAT, and H1734 cells (The Human Integrated Protein Expression Database, Integrated protein expression data from ProteomicsDB, PaxDb, MaxQB, and MOPED).

**Figure 1:**
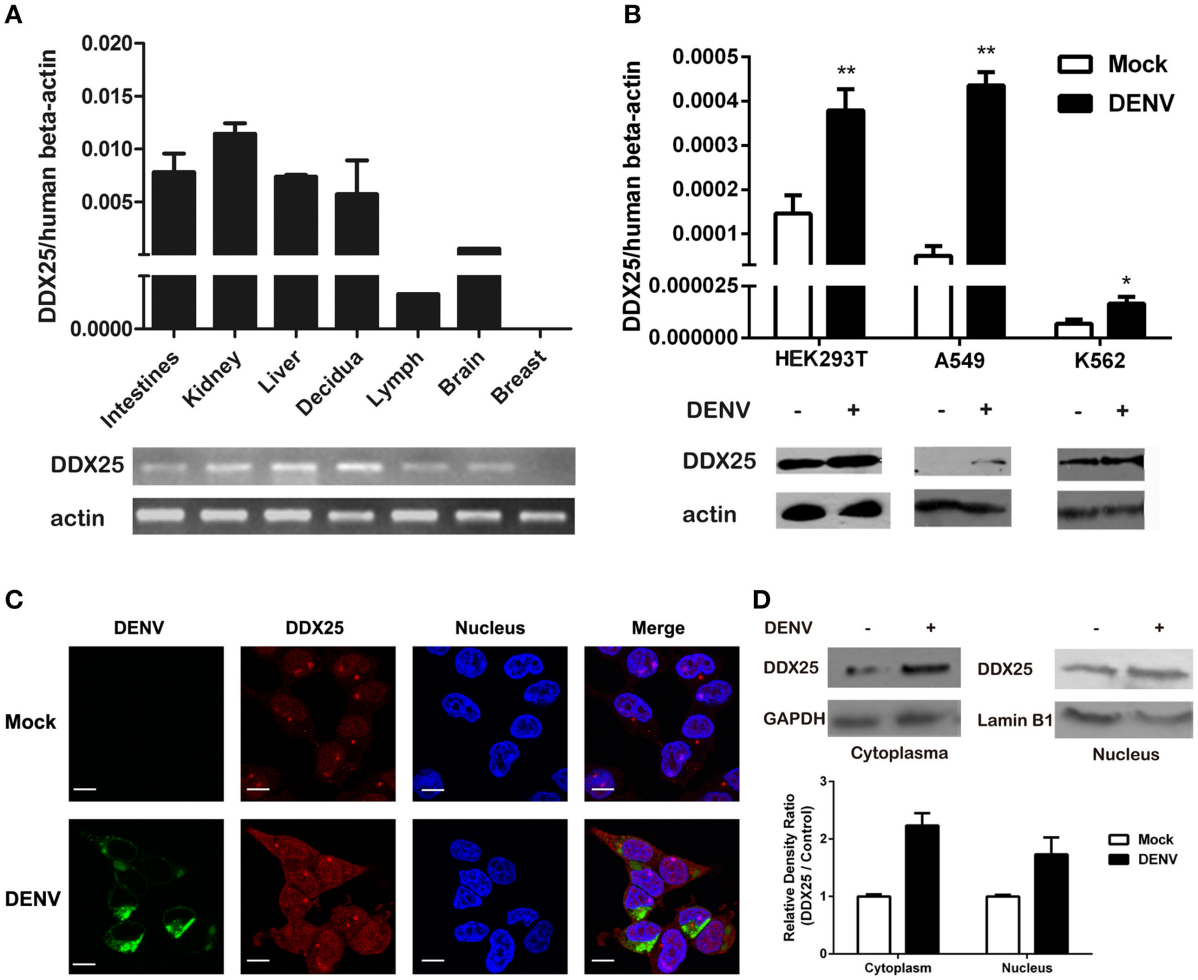
Human DDX25 is ubiquitously expressed and upregulated after DENV infection. **(A)** qRT-PCR and electrophoresis analysis of *DDX25* expression in various human tissues. Human β*-actin* was used as an internal control. **(B)** The qRT-PCR and immunoblots analysis of DDX25 expression in HEK293T, A549, and K562 cells before and after DENV infection. **(C)** Microscopic images of immunofluorescence staining for expression and subcellular distribution of DDX25 in HEK293T with or without DENV infection. (MOI of 1 for 24 h). Scale bars, 10 μm. **(D)** Immunoblots analysis of DDX25 expression in the cytoplasm and nucleus before and after DENV infection. The relative gray density of immunoblots bands (DDX25/β-actin) was analyzed by ImageJ software. Results were expressed as the mean + the SEM. ^*^p < 0.05 and ^**^p < 0.01 (*t*-test). Representative results from at least three independent experiments.

We further confirmed the mRNA expression profile of human *DDX25* gene by qRT-PCR. As shown in Figure 1A, *DDX25* mRNA is expressed in a wide range of human tissues. With the exception of breast tissue, *DDX25* is expressed in all seven tested tissues, with the highest expression in the kidney (Figure 1A).

We further noted an increased *DDX25* expression in human cell line HEK293T, A549, and K562 during DENV infection. Consistently, the protein level of DDX25 was also significantly induced in cells infected with DENV (Figure 1B). We further investigated the cellular distribution of DDX25 in HEK293T cells before and after DENV infection. Interestingly, DDX25 expression was upregulated both in cytoplasm and nucleus (Figures 1C,D). These data demonstrate that DDX25 may have a low basal level of expression in multiple tissues and cells, and can be upregulated upon DENV infection.

### DDX25 promotes virus replication

**Figure 2:**
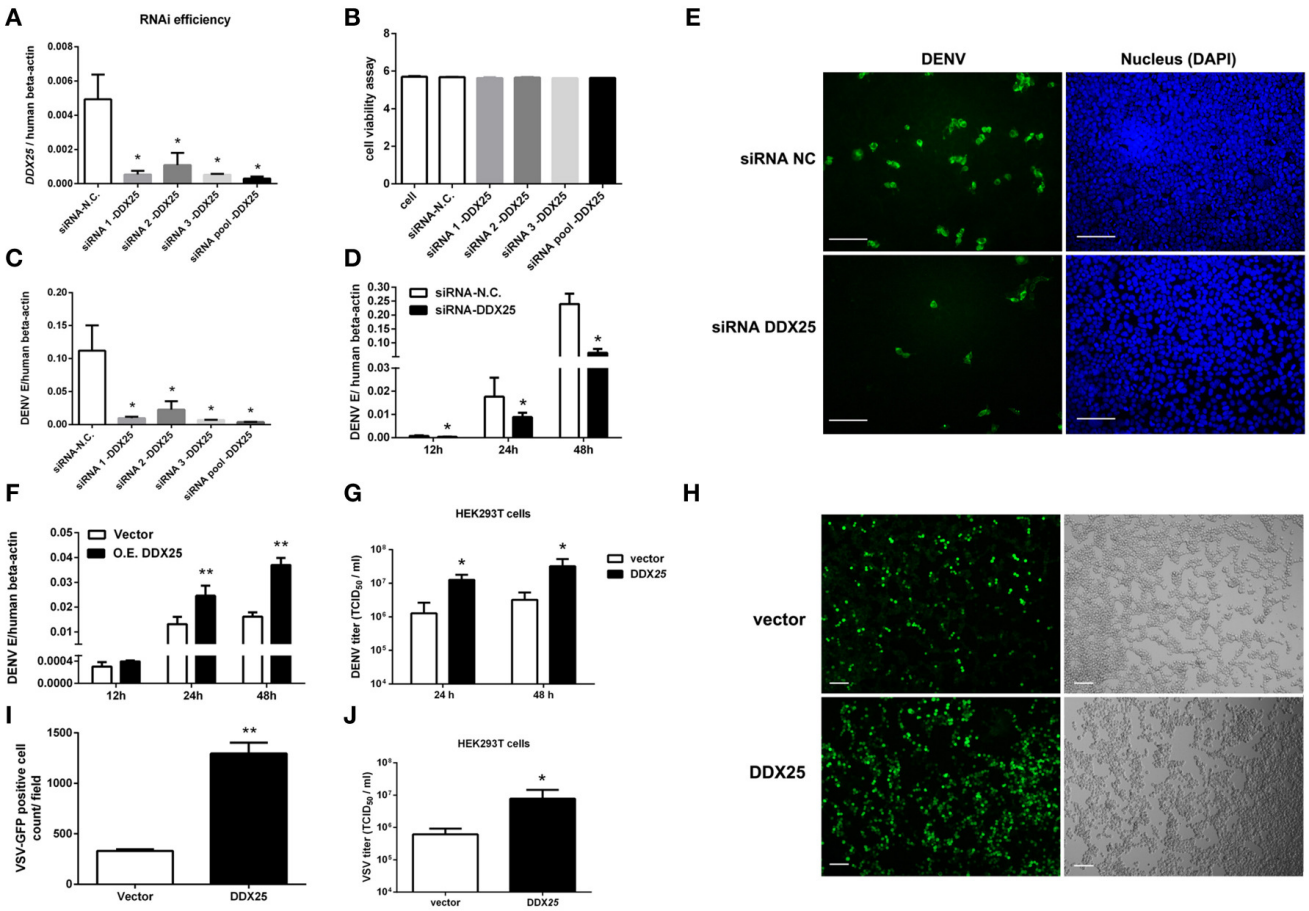
DDX25 promotes virus replication. **(A)** The RNAi efficiency of three individual or pooled siRNAs that targeting *DDX25* were tested in transfected HEK293T cells by qRT-PCR. **(B)** RNAi of *DDX25* showed no cytotoxic effect to HEK 293T cells (Measured by Cell Viability assay, Promega). **(C)** DENV viral loads in *DDX25* siRNAs (or NC siRNA) treated HEK293T cells at 24 h post DENV infection (MOI of 1). The viral burdens were analyzed by measuring the virus E gene copy using qRT-PCR, and normalized to human β*-actin*. **(D)** DENV viral loads in *DDX25* siRNA (or NC siRNA) treated HEK293T cells at 12, 24, and 48 h post DENV infection (MOI of 1). **(E)** Microscopic images of immunofluorescence staining for DENV E antigen in *DDX25* siRNA (or NC siRNA) treated HEK293T after DENV infection for 24 h (MOI of 1). Scale bars, 100 μm. **(F)** qRT-PCR analysis of DENV viral loads in HEK293T cells with or without DDX25 overexpression at 12, 24, and 48 h post DENV infection. **(G)** Viral titers in supernatants of DENV infected HEK293T cells determined by TCID_50_ assay on Vero cells. **(H,I)** Fluorescence microscopy analysis of VSV-GFP infection of HEK293T cells transfected with vector or DDX25 plasmid. Viral infection rates were indicated with GFP signal and quantified with ImageJ software. Scale bars, 100 μm. **(J)** VSV titers in supernatants of HEK293T cells infected with VSV-GFP (MOI of 1) at 24 h. Results were expressed as the mean + the SEM. ^*^p < 0.05 and ^**^p < 0.01 (*t*-test). Representative results from at least three independent experiments.

Our previous studies of RNAi screening against DDX family demonstrated that DDX25 could be a proviral factor for DENV infection in HEK293T cells (Li G. et al., 2015). To confirm the role of endogenous DDX25 in DENV replication, endogenous DDX25 expression was silenced using RNAi approach. SiRNAs that targeting *DDX25* significantly reduced *DDX25* mRNA expression compared with cells transfected with scramble siRNA (N.C.) (Figure 2A). Cell viability was not affected after siRNA transfection, as tested through Promega Cell Viability Assay (Figure 2B). Silencing *DDX25* with three individual or pooled siRNAs significantly impaired DENV replication at 24 h post-infection (Figure 2C) [The intracellular viral loads were determined by measuring the transcript levels of the DENV envelop gene (E) and normalized to human β*-actin*]. Further experiment confirmed that the DENV viral load were decreased by ~2 to 4-fold (*p* < 0.05) in *DDX25*-silenced cells compared with control cells after DENV infection at 12, 24, and 48 h, respectively (Figure 2D). Similar results were observed in *DDX25* silenced K562 cells infected with DENV (Data not shown). In line with this, immunofluorescence assay showed DENV viral burden [as determined by staining of viral envelop (E) protein] were decreased in *DDX25* silenced cells (Figure 2E). Conversely, we noted a ~4-fold increase in viral load in *DDX25* overexpressing HEK293T cell at 48 h post-infection (Figure 2F). Consistently, the titers of DENV in cell supernatants, as determined by TCID_50_ assay, were significantly higher in DDX25 overexpressed cells (Figure 2G). Moreover, we observed elevated viral loads in DDX25 overexpressing HEK293T cells after infection with VSV-GFP virus (Figures 2H–J). Overall, these data suggest that DDX25 could promote DENV and VSV replication.

### DDX25 regulates IFNβ production

**Figure 3:**
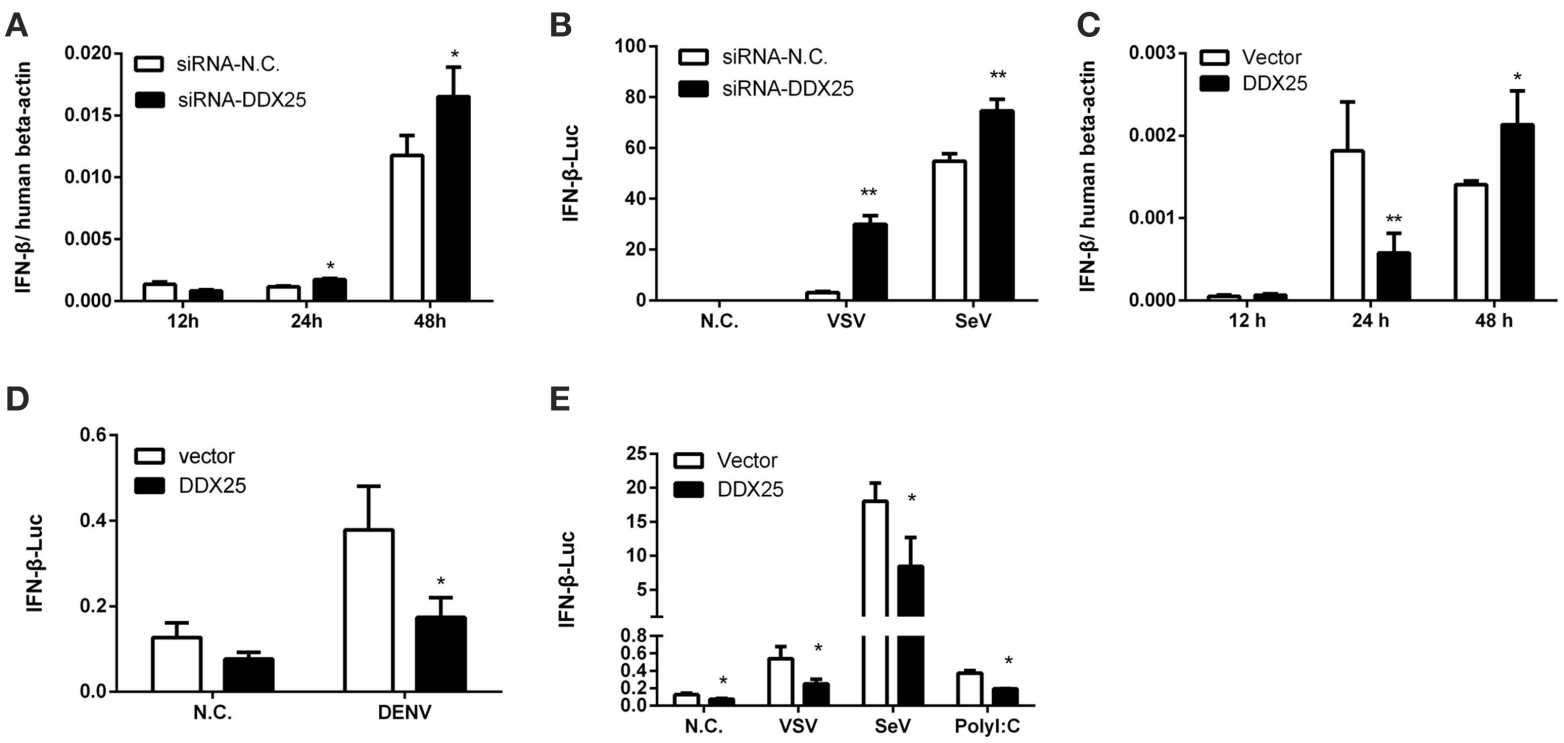
DDX25 negatively regulates IFNβ production. **(A)** qRT-PCR analysis of the mRNA levels of IFNβ in HEK293T cells with *DDX25* RNAi during DENV infection for 12, 24, and 48 h. **(B)** VSV and SeV induced IFNβ transcriptional activities were enhanced in *DDX25* silenced HEK293T cells compared to controls. **(C)** qRT-PCR analysis of the mRNA levels of *IFN*β in control and *DDX25* overexpressed HEK293T cells during DENV infection for 12, 24, and 48 h. **(D,E)** Luciferase reporter assay of IFNβ transcriptional activity in *DDX25* overexpressed HEK293T cells during DENV **(D)**, VSV, SeV, and Poly I:C **(E)** stimulation. Results were expressed as the mean + the SEM. ^*^p < 0.05 and ^**^p < 0.01 (*t*-test). Representative results from at least three independent experiments.

The aforementioned results clearly demonstrate the importance of DDX25 in promoting DENV infection *in vitro*. We next evaluated whether DDX25 influence the immune response to viral infection. The mRNA expression of *IFN*β was increased by 1.5- and 1.4-fold at 24 and 48 h post DENV infection respectively, in *DDX25* silenced cells compared with controls (Figure 3A). *DDX25* silencing also enhanced the IFNβ promoter-driven luciferase (IFNβ-Luc) expression in HEK293T cells infected with other RNA viruses, such as VSV and sendai virus (SeV) (Figure 3B). Conversely, we noted a decreased *IFN*β mRNA level on 24 h in DDX25-overexpressed HEK293T cells post DENV infection (Figure 3C). While, at 48 h post-infection, the *IFN*β mRNA level was increased in DDX25 overexpressed cells (Figure 3C). We hypothesized that this was due to the expansion of DENV burdens in those cells at the late time point, which induced more IFNs that DDX25 could not fully suppressed. Consistent with the qPCR results, overexpression of DDX25 could decrease the transcription of IFNβ-Luc reporter after DENV (Figure 3D) and other RNA virus infection (Figure 3E). These results suggest that DDX25 negatively regulates innate immune-signaling processes and suppress type I IFN production during virus infection.

### DDX25 transgenic mice were more susceptible to RNA virus infection

**Figure 4:**
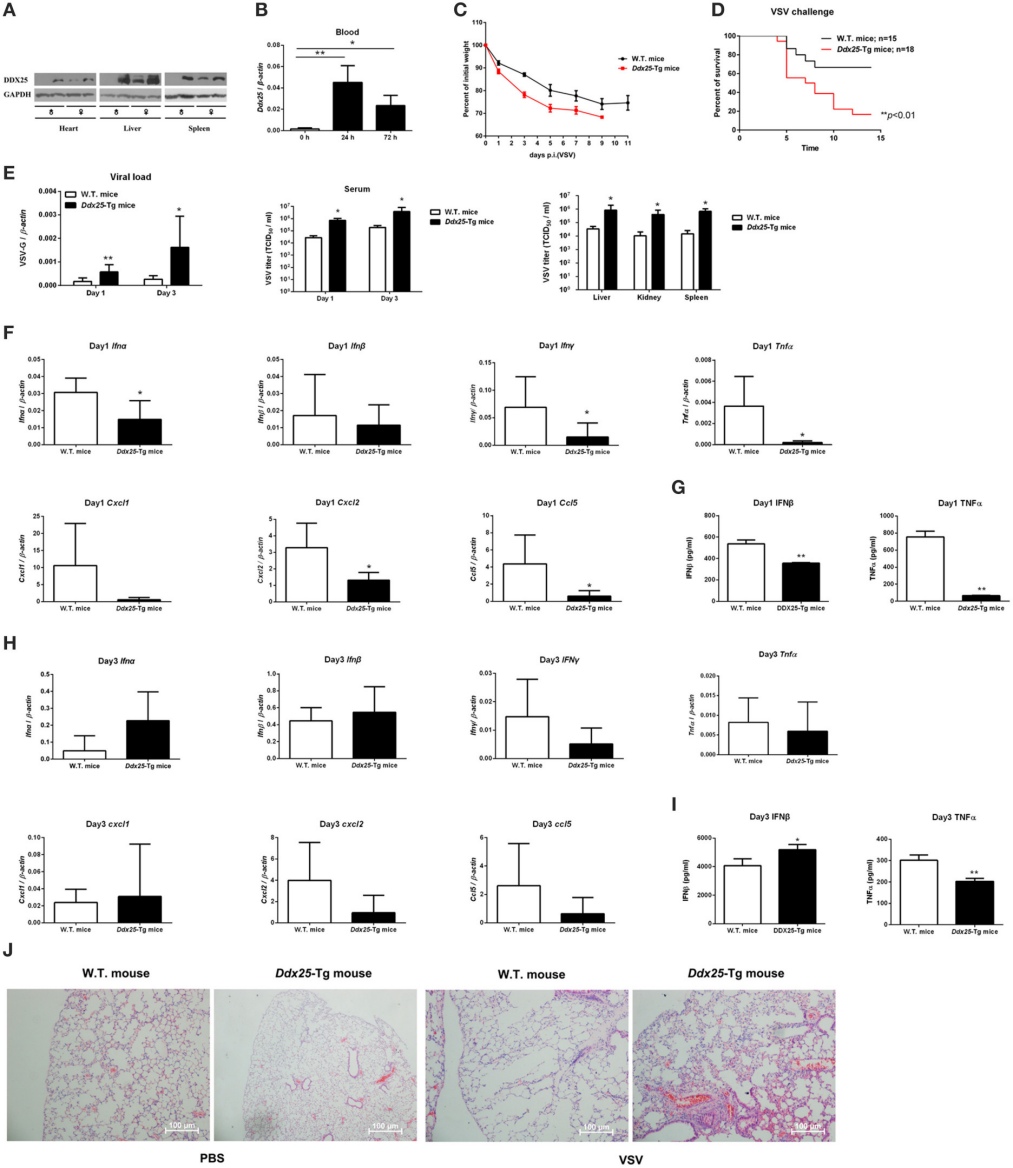
Exacerbated VSV pathogenesis in *Ddx25* transgenic mice. Seven to eight weeks old, sex-matched, C57BL/6 (W.T.), and *Ddx25*-Tg mice were challenged with VSV (1 × 10^6^ *pfu*/mouse). **(A)** Immunoblots of DDX25 in various tissues of W.T. and *Ddx25*-Tg mice, suggesting the evaluated expression of DDX25 in *Ddx25*-Tg mice. **(B)** qRT-PCR quantification of *Ddx25* mRNA in whole blood cells of wild type (W.T.) mice during VSV infection for 0, 24, and 72 h. **(C,D)** Changes in body weight **(C)** and survival rate **(D)** of W.T., *Ddx25*-Tg mice infected VSV, as monitored daily until days 11 and 14 post-infection, respectively. **(E)** VSV titers in the blood and tissue lysates of liver, kidney, and spleen from infected mice were measured via qRT-PCR and TCID_50_ assay. (**F–I**) The expression levels of the selective cytokines/chemokines *(Ifn*α, *Ifn*β, *Ifn*γ, *Cxcl1, Cxcl2, Ccl5*, and *Tnf*α) in total leukocytes of W.T. and *Ddx25*-Tg mice (*N* = 10 each) at day 1 **(F,G)** and day 3 **(H,I)** post-infection. Expression levels of mRNAs were determined by qRT-PCRs **(F,H)**. Protein levels of IFNβ and TNFα in serums at day 1 **(G)** and day 3 **(I)** were determined by ELISA. Results were expressed as the mean + the SEM. ^*^p < 0.05 and ^**^p < 0.01 (*t*-test). Representative results from at least three independent experiments. **(J)** H&E staining of lung tissues of W.T. and *Ddx25*-Tg mice at 2 days post mock infection (PBS) or VSV infection. Scale bars, 100 μm.

DDX25 null mice are sterile due to a defect in spermiogenesis (Tsai-Morris et al., 2004). So we constructed *Ddx25*-Tg mice that overexpress DDX25 to identify the role of *Ddx25* in virus infection *in vivo* (Figure 4A). Since immunocompetent wild-type mice do not show dengue hemorrhagic fever-like symptoms upon DENV infection, we used a VSV-infection mouse model to evaluate the importance of DDX25 in RNA viral infection *in vivo*. Consistent with the *in vitro* data, the *Ddx25* mRNA in total blood cells was upregulated after VSV virus infection in wild type mice (Figure 4B). Wild-type (W.T.) and *Ddx25*-Tg mice were infected intranasally with a lethal dose of VSV (1 × 10^6^ *pfu*). All infected *Ddx25*-Tg mice show increased body weight loss compared with wide type controls (Figure 4C). Approximately 90% of *Ddx25*-Tg mice died within 10 days post-infection (Figure 4D), while only 40% of the wide type control mice died due to the VSV infection. This suggested that *Ddx25*-Tg mice are more susceptible to VSV infection. The viremia, in terms of the copy number of VSV glycoprotein gene (VSV-G) in blood cells and infectious viral particles in serum, were significantly higher in *Ddx25*-Tg mice compared with W.T. mice at day 1 and day 3 after infection. We also found that VSV titers were higher in the liver, kidney, and spleen of *Ddx25*-Tg mice than in those organs of W.T. mice (Figure 4E). Meanwhile, the mRNA expression of *Ifn*α, *Ifn*β, *Ifn*γ*, Tnf*α, and selective chemokines (*Cxcl1, Cxcl2*, and *Ccl5*) by leukocytes was lower in the blood of *Ddx25*-Tg mice on day 1 post-infection (Figure 4F). The IFNβ and TNFα proteins in the serum were also significantly lower in *Ddx25*-Tg mice compared with those in controls (Figure 4G). While, *Ifn*α and *Ifn*β expression on day 3 post-infection were increased in *Ddx25*-Tg mice, this may be the secondary effect of the increased viremia (Figure 4H). The protein levels of IFNβ and TNFα were consistent with their mRNA levels (Figure 4I). Histological analysis by H&E staining indicated that the bronchial lumen and alveolar both exhibit healthy integrity in the non-infected W.T. and *Ddx25*-Tg mice. However, the W.T. mice infected virus displayed mucosal edema, inflammatory cell infiltration (submucosal and mucosal layers), as well as the increased number of eosinophils (EOS) in the airway walls. Compared to the virus infected W.T. mice group, a significantly increased inflammatory cell infiltration and serious immunopathological tissue damage were observed in VSV infected *Ddx25*-Tg mice (Figure 4J). These data suggested that DDX25 could promote RNA virus infection and suppress *Ifn*β and other cytokines production *in vivo*.

### DDX25 inhibits IFN production by interrupting IRF3 and NFκB activation

**Figure 5:**
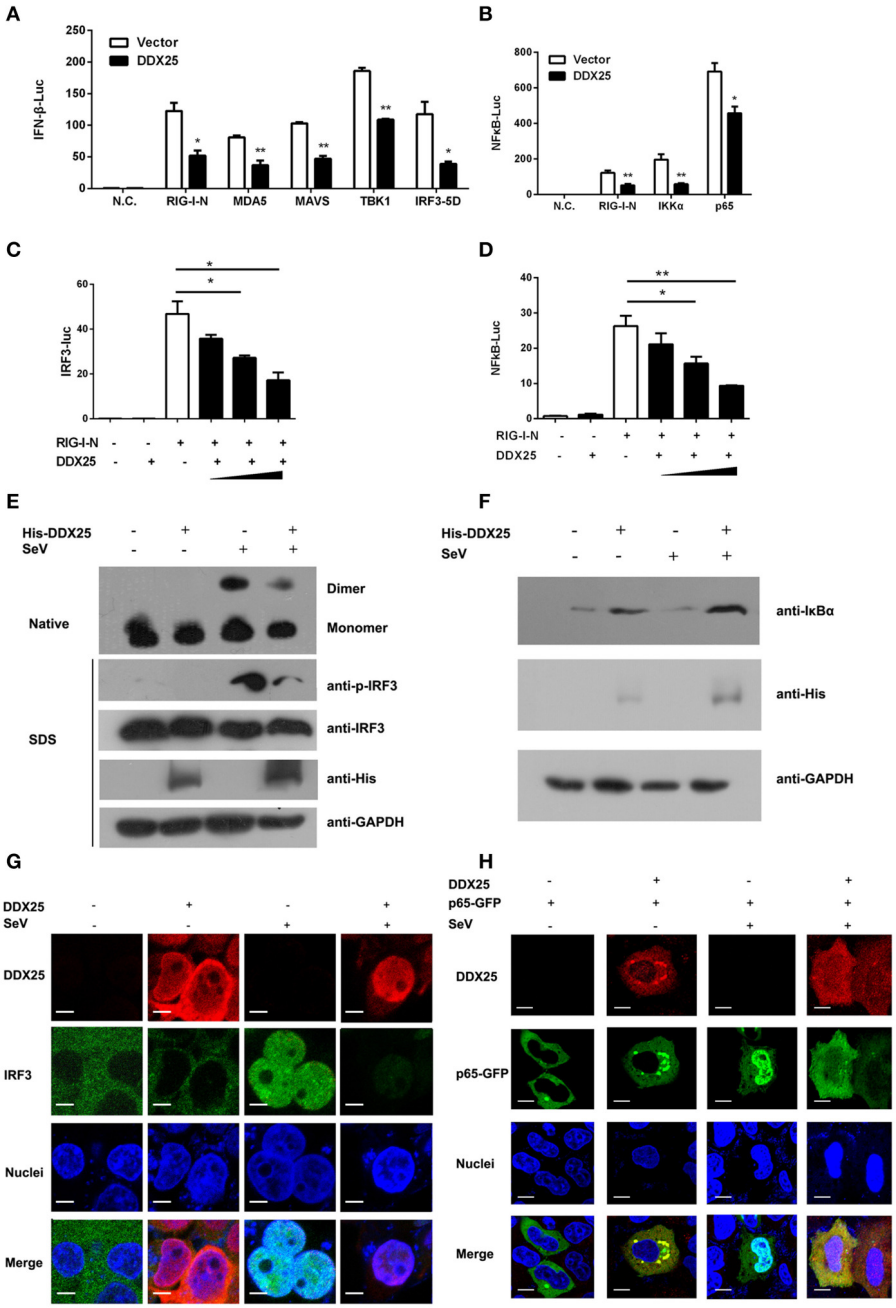
DDX25 interrupts the IFN-signaling pathway by blocking IRF3 and NFκB activation. **(A)** Overexpression of DDX25 inhibits RIG-IN, MDA-5, MAVS, TBK1, and IRF3-5D directed IFNβ promoter activation. IFNβ-Luc assay was performed in vector or DDX25 transfected HEK293T cells. IFNβ promoter activations were driven by co-expression of distinct RIG-I signaling pathway molecules. **(B)** Overexpression of DDX25 inhibits IG-IN, IKKα, and NFκB p65 induced NFκB promoter activation. NFκB promoter was activated by transfecting RIG-IN, IKKα, or NFκB p65, and measured by a NFκB-Luc. **(C,D)** DDX25 suppresses RIG-IN directed IRF3-Luc **(C)** and NFκB-Luc **(D)** in a dose dependent manner. Results are expressed as the mean + the SEM. ^*^p < 0.05 and ^**^p < 0.01 (*t*-test). Representative results from at least three independent experiments. **(E)** Overexpression of DDX25 impaired SeV induced IRF3 phosphorylation and dimerization. **(F)** Overexpression of DDX25 suppressed SeV induced IκB degradation. **(G,H)** Nucleic translocation of IRF3 **(G)** and NFκB p65 **(H)** in SeV stimulated HEK293T cells were impaired by DDX25. Immunofluorescence assay were performed in control or DDX25 overexpressed HEK293T cells with or without SeV infection. IRF3, DDX25 were probed with specific primary antibodies followed by TRITC or FITC labeled secondary antibodies. NFκB p65 was visualized by a fused GFP protein. Scale bars, 10 μm.

We next sought to understand how DDX25 suppresses of IFNβ induction. Particularly, RNA recognition by RIG-I emanates an activation signal that is passed on sequentially to mitochondrial antiviral signaling protein (MAVS), TBK1, IKKε, IRF3, and IRF7 (Sharma et al., 2003; Liu et al., 2015; Yoneyama et al., 2015). Therefore, the action point of DDX25 can be determined by assessing its suppressive effects on a series of transducer proteins. As the first step, we chose to evaluate the impact of DDX25 on the IFN-inducing activity of RIG-I-signaling pathway components, including the active caspase recruitment domain (CARD) containing form of RIG-I (RIG-IN), MDA5, MAVS, TBK1 kinase, and the active form of IRF3 (IRF3/5D) (Lin et al., 1998), or IKKα and p65. DDX25-expressing plasmids, together with one of the IFN pathway activators, RIG-IN, MDA5, MAVS, TBK1, IRF3/5D, IKKα, or p65, were co-transfected into HEK293T cells. All the expression constructs of IFN pathway activators induced an IFNβ-Luc or NFκB-Luc reporter activity. Interestingly, DDX25 was found to potently inhibit IFNβ-Luc activity induced by RIG-IN, MDA5, MAVS, TBK1, and IRF3/5D (Figure 5A), as well as NFκB-Luc reporter activity induced by RIG-IN, IKKα and p65 (Figure 5B). These results indicate that DDX25 inhibits the IFN antiviral response at or downstream of IRF3 and NFκB.

To further validate the step in which DDX25 inhibits IRF3 and NFκB activation, IRF3 and NFκB-specific luciferases activity were induced by coexpression of RIG-IN. In the presence of DDX25, the IRF3 and NFκB activities induced by RIG-IN were reduced in a DDX25 dose-dependent manner (Figures 5C,D), which imply that DDX25 inhibits both IRF3 and NFκB transcriptional activity directly.

IRF3 and NFκB activation requires the association of distinct signaling molecules (Baeuerle and Baichwal, 1997; Barnes and Karin, 1997). Phosphorylation of IRF3 induces a conformational change, leading to IRF3 dimerization and nuclear translocation. While activation of the NFκB is initiated by the signal-induced degradation of IκB proteins. IκB proteins mask the nuclear localization signals (NLS) of NFκB proteins and keep them sequestered in an inactive state in the cytoplasm of unstimulated cells. To investigate whether IRF3 and NFκB activation were directly or indirectly affected by DDX25, we examined the protein level of IκBα and phosphorylation of IRF3 in the DDX25 overexpressed cells. Western blot results revealed that SeV induced the phosphorylation of IRF3 and the degradation of IκBα was inhibited by DDX25 (Figures 5E,F). Complementary to phosphorylation, SeV infection induced the dimerization of IRF3 and is also inhibited by DDX25 overexpression (Figure 5E). These results suggest that DDX25 inhibits IRF3 phosphorylation and NFκB activation.

To strengthen our existing data, immunofluorescence was carried out to investigate whether DDX25 prevented the nuclear translocation of IRF3 and NFκB p65 subunit. In mock-treated HeLa cells, IRF3, and p65 localized exclusively to the cytoplasm. SeV infection induced nuclear translocation of IRF3 and p65 in cells, while ectopic expression of DDX25 impaired the nuclear translocation of IRF3 and p65 (Figures 5G,H). Taken together, these results indicate that DDX25 inhibits the host antiviral response, by repressing the IFN antiviral response by suppressing IRF3 and NFκB transcriptional activity, and thereby inhibited the host antiviral response.

## Discussion

Previous work suggested that GRTH/DDX25 is highly expressed in testis and essential for completion of spermatogenesis in mouse mode (Mendelson, 2013). In this report, we further suggested that DDX25 is expressed in multiple human tissues and cell lines. Strikingly, we observed an increased production of DDX25 both *in vivo* and *in vitro* during viral infection. We further discovered DDX25 promotes the replication of RNA viruses, including DENV, VSV (Figures 2G,H), and Zika virus (ZIKV) (data not shown). *In vivo* data also demonstrates that DDX25 promotes RNA virus replication. As such, DDX25 has additional function for viral infection in human cells and mouse models.

The IFN system is triggered after viral infection via cellular recognition of viral components. Mammalian cells utilize multiple pattern recognition receptors, e.g., TLRs, RLRs, NLRs, or cGAS/STING, to detect the incoming viral particles in the cytoplasm or inside specific cellular compartments and trigger intracellular signaling pathways, which lead to the induction of IFN through the action of the transcription factor IRF3 (Chattopadhyay and Sen, 2016). Viral infection activates IRF3 by causing phosphorylation of its specific serine residues and its translocation to the nucleus, where it binds to the promoters of the target genes (Lin et al., 1998; Sato et al., 1998). On the other hand, NFκB is rapidly activated after exposure to pathogens (Rahman and McFadden, 2011). Once IκB is degraded, the NFκB complex is translocated to the nucleus where it functions as a transcription factor for numerous effector genes including type I IFN. Our data here indicated that DDX25 may negatively regulate these processes. Recently, the DEAD/H-box helicase family members have been drawing more and more attention not only for their powerful capacities in detecting invading pathogen-associated molecular patterns (PAMPs) as direct sensors (Fullam and Schroder, 2013; Mitoma et al., 2013), but also for their key roles involved in viral replication (Wang et al., 2009; Fullam and Schroder, 2013; Yasuda-Inoue et al., 2013; Zhou et al., 2013). RIG-I (DDX58) is reported to be a key sensor for viral RNA mediated innate immune signaling processes (Schmidt et al., 2012). Several other RNA helicases, such as DDX19 (Li J. et al., 2015), DDX41 (Jiang et al., 2017), DHX9 and DHX36 (Kim et al., 2010), are also implicated in the regulation of host defense processes. DDX3X and DDX24 function downstream of nucleic acid recognition to affect multi-protein signaling complexes required for efficient primary innate immune gene transcription (Ma et al., 2013; Li G. et al., 2015).

To evade the antiviral response of host cells, many viruses utilize the host factors to interfere with IRF3 or NFκB signaling by sophisticated mechanisms (Rahman and McFadden, 2011; Ye et al., 2013). Here, we demonstrate that DDX25 interfered with the activation of IRF3 and NFκB induced by DENV and other RNA viruses. Our findings suggested that DDX25 alters the phosphorylation level of IRF3 and the degradation of IκBα mediated by SeV.

DDX25 plays a negative regulatory role for IFNβ during DENV replication, confirming that inhibition of nuclear-cytoplasmic protein transport is a strategy for a virus to prevent the antiviral response of host cells. In summary, our data demonstrated that DDX25 interferes with the nuclear translocation of IRF3 and NFκB induced by RNA virus. Via this mechanism, DDX25 suppresses the induction of type I IFN in host cells during viral infection. These findings reveal a novel strategy for DENV to utilize a host factor to evade the host innate immune response and provide us new insight into the function of DDX25.

## Author contributions

TF and JD designed the experiments and analyzed the data. TF, TS, GL, KW, and WP performed the experiments. TF and JD wrote the manuscript with all the authors contributing to writing, discussion, and agreeing with the conclusion presented in the manuscript.

### Conflict of interest statement

The authors declare that the research was conducted in the absence of any commercial or financial relationships that could be construed as a potential conflict of interest.

## References

[B1] Ariumi Y. Kuroki M. Abe K. Dansako H. Ikeda M. Wakita T.. (2007). DDX3 DEAD-box RNA helicase is required for hepatitis C virus RNA replication. J. Virol. 81, 13922–13926. 10.1128/JVI.01517-07 17855521 PMC2168844

[B2] Baeuerle P. A. Baichwal V. R. (1997). NF-kappa B as a frequent target for immunosuppressive and anti-inflammatory molecules. Adv. Immunol. 65, 111–137. 10.1016/S0065-2776(08)60742-7 9238509

[B3] Barnes P. J. Karin M. (1997). Nuclear factor-kappaB: a pivotal transcription factor in chronic inflammatory diseases. N. Engl. J. Med. 336, 1066–1071. 10.1056/NEJM199704103361506 9091804

[B4] Chahar H. S. Chen S. Manjunath N. (2013). P-body components LSM1, GW182, DDX3, DDX6 and XRN1 are recruited to WNV replication sites and positively regulate viral replication. Virology 436, 1–7. 10.1016/j.virol.2012.09.041 23102969 PMC3545066

[B5] Chattopadhyay S. Sen G. C. (2016). RIG-I-like receptor-induced IRF3 mediated pathway of apoptosis (RIPA): a new antiviral pathway. Protein Cell 8, 165–168. 10.1007/s13238-016-0334-x 27815826 PMC5326620

[B6] de la Cruz J. Kressler D. Linder P. (1999). Unwinding RNA in *Saccharomyces cerevisiae*: DEAD-box proteins and related families. Trends Biochem. Sci. 24, 192–198. 10.1016/S0968-0004(99)01376-6 10322435

[B7] Fang J. Kubota S. Yang B. Zhou N. Zhang H. Godbout R.. (2004). A DEAD box protein facilitates HIV-1 replication as a cellular co-factor of Rev. Virology 330, 471–480. 10.1016/j.virol.2004.09.039 15567440

[B8] Fullam A. Schroder M. (2013). DExD/H-box RNA helicases as mediators of anti-viral innate immunity and essential host factors for viral replication. Biochim. Biophys. Acta 1829, 854–865. 10.1016/j.bbagrm.2013.03.012 23567047 PMC7157912

[B9] Gan V. C. (2014). Dengue: moving from current standard of care to state-of-the-art treatment. Curr. Treat. Options Infect. Dis. 6, 208–226. 10.1007/s40506-014-0025-1 25999799 PMC4431705

[B10] Guzman M. G. Harris E. (2015). Dengue. Lancet 385, 453–465. 10.1016/S0140-6736(14)60572-9 25230594

[B11] Jankowsky E. (2010). RNA helicases at work: binding and rearranging. Trends Biochem. Sci. 36, 19–29. 10.1016/j.tibs.2010.07.008 20813532 PMC3017212

[B12] Jiang Y. Zhu Y. Liu Z. J. Ouyang S. (2017). The emerging roles of the DDX41 protein in immunity and diseases. Protein Cell 8, 83–89. 10.1007/s13238-016-0303-4 27502187 PMC5291771

[B13] Kavarthapu R. Dufau M. L. (2015). Germ cell nuclear factor (GCNF/RTR) regulates transcription of gonadotropin-regulated testicular RNA helicase (GRTH/DDX25) in testicular germ cells–the androgen connection. Mol. Endocrinol. 29, 1792–1804. 10.1210/me.2015-1198 26484580 PMC4664233

[B14] Khadka S. Vangeloff A. D. Zhang C. Siddavatam P. Heaton N. S. Wang L.. (2011). A physical interaction network of dengue virus and human proteins. Mol. Cell. Proteomics 10:M111.012187. 10.1074/mcp.M111.012187 21911577 PMC3237087

[B15] Kim T. Pazhoor S. Bao M. Zhang Z. Hanabuchi S. Facchinetti V.. (2010). Aspartate-glutamate-alanine-histidine box motif (DEAH)/RNA helicase A helicases sense microbial DNA in human plasmacytoid dendritic cells. Proc. Natl. Acad. Sci. U.S.A. 107, 15181–15186. 10.1073/pnas.1006539107 20696886 PMC2930588

[B16] Li G. Feng T. Pan W. Shi X. Dai J. (2015). DEAD-box RNA helicase DDX3X inhibits DENV replication via regulating type one interferon pathway. Biochem. Biophys. Res. Commun. 456, 327–332. 10.1016/j.bbrc.2014.11.080 25437271

[B17] Li J. Hu L. Liu Y. Huang L. Mu Y. Cai X.. (2015). DDX19A senses viral RNA and mediates NLRP3-dependent inflammasome activation. J. Immunol. 195, 5732–5749. 10.4049/jimmunol.1501606 26538395

[B18] Lin R. Heylbroeck C. Pitha P. M. Hiscott J. (1998). Virus-dependent phosphorylation of the IRF-3 transcription factor regulates nuclear translocation, transactivation potential, and proteasome-mediated degradation. Mol. Cell. Biol. 18, 2986–2996. 10.1128/MCB.18.5.2986 9566918 PMC110678

[B19] Liu S. Cai X. Wu J. Cong Q. Chen X. Li T.. (2015). Phosphorylation of innate immune adaptor proteins MAVS, STING, and TRIF induces IRF3 activation. Science 347:aaa2630. 10.1126/science.aaa2630 25636800

[B20] Ma Z. Moore R. Xu X. Barber G. N. (2013). DDX24 negatively regulates cytosolic RNA-mediated innate immune signaling. PLoS Pathog. 9:e1003721. 10.1371/journal.ppat.1003721 24204270 PMC3814876

[B21] Mendelson C. R. (2013). GRTH: a key to understanding androgen-mediated germ cell signaling. Endocrinology 154, 1967–1969. 10.1210/en.2013-1395 23687113 PMC3740485

[B22] Mitoma H. Hanabuchi S. Kim T. Bao M. Zhang Z. Sugimoto N.. (2013). The DHX33 RNA helicase senses cytosolic RNA and activates the NLRP3 inflammasome. Immunity 39, 123–135. 10.1016/j.immuni.2013.07.001 23871209 PMC3756931

[B23] Parvatiyar K. Zhang Z. Teles R. M. Ouyang S. Jiang Y. Iyer S. S.. (2012). The helicase DDX41 recognizes the bacterial secondary messengers cyclic di-GMP and cyclic di-AMP to activate a type I interferon immune response. Nat. Immunol. 13, 1155–1161. 10.1038/ni.2460 23142775 PMC3501571

[B24] Rahman M. M. McFadden G. (2011). Modulation of NF-kappaB signalling by microbial pathogens. Nat. Rev. Microbiol. 9, 291–306. 10.1038/nrmicro2539 21383764 PMC3611960

[B25] Rocak S. Linder P. (2004). DEAD-box proteins: the driving forces behind RNA metabolism. Nat. Rev. Mol. Cell Biol. 5, 232–241. 10.1038/nrm1335 14991003

[B26] Sato M. Tanaka N. Hata N. Oda E. Taniguchi T. (1998). Involvement of the IRF family transcription factor IRF-3 in virus-induced activation of the IFN-beta gene. FEBS Lett. 425, 112–116. 10.1016/S0014-5793(98)00210-5 9541017

[B27] Schmidt A. Rothenfusser S. Hopfner K. P. (2012). Sensing of viral nucleic acids by RIG-I: from translocation to translation. Eur. J. Cell Biol. 91, 78–85. 10.1016/j.ejcb.2011.01.015 21496944 PMC3155743

[B28] Sharma S. tenOever B. R. Grandvaux N. Zhou G. P. Lin R. Hiscott J. (2003). Triggering the interferon antiviral response through an IKK-related pathway. Science 300, 1148–1151. 10.1126/science.1081315 12702806

[B29] Sheng Y. Tsai-Morris C. H. Dufau M. L. (2003). Cell-specific and hormone-regulated expression of gonadotropin-regulated testicular RNA helicase gene (GRTH/Ddx25) resulting from alternative utilization of translation initiation codons in the rat testis. J. Biol. Chem. 278, 27796–27803. 10.1074/jbc.M302411200 12734186

[B30] Steimer L. Klostermeier D. (2012). RNA helicases in infection and disease. RNA Biol. 9, 751–771. 10.4161/rna.20090 22699555

[B31] Thisyakorn U. Thisyakorn C. (2014). Latest developments and future directions in dengue vaccines. Ther. Adv. Vaccines 2, 3–9. 10.1177/2051013613507862 24757522 PMC3991153

[B32] Tsai-Morris C. H. Sheng Y. Lee E. Lei K. J. Dufau M. L. (2004). Gonadotropin-regulated testicular RNA helicase (GRTH/Ddx25) is essential for spermatid development and completion of spermatogenesis. Proc. Natl. Acad. Sci. U.S.A. 101, 6373–6378. 10.1073/pnas.0401855101 15096601 PMC404052

[B33] Wang H. Kim S. Ryu W. S. (2009). DDX3 DEAD-Box RNA helicase inhibits hepatitis B virus reverse transcription by incorporation into nucleocapsids. J. Virol. 83, 5815–5824. 10.1128/JVI.00011-09 19297497 PMC2681949

[B34] Wang K. Wang J. Sun T. Bian G. Pan W. Feng T.. (2016). Glycosphingolipid GM3 is indispensable for dengue virus genome replication. Int. J. Biol. Sci. 12 872–883. 10.7150/ijbs.15641 27313500 PMC4910605

[B35] Yasuda-Inoue M. Kuroki M. Ariumi Y. (2013). Distinct DDX DEAD-box RNA helicases cooperate to modulate the HIV-1 Rev function. Biochem. Biophys. Res. Commun. 434, 803–808. 10.1016/j.bbrc.2013.04.016 23608157 PMC7092829

[B36] Ye J. Zhu B. Fu Z. F. Chen H. Cao S. (2013). Immune evasion strategies of flaviviruses. Vaccine 31 461–471. 10.1016/j.vaccine.2012.11.015 23153447

[B37] Yoneyama M. Onomoto K. Jogi M. Akaboshi T. Fujita T. (2015). Viral RNA detection by RIG-I-like receptors. Curr. Opin. Immunol. 32, 48–53. 10.1016/j.coi.2014.12.012 25594890

[B38] Zhang Z. Kim T. Bao M. Facchinetti V. Jung S. Y. Ghaffari A. A.. (2011a). DDX1, DDX21, and DHX36 helicases form a complex with the adaptor molecule TRIF to sense dsRNA in dendritic cells. Immunity 34, 866–878. 10.1016/j.immuni.2011.03.027 21703541 PMC3652560

[B39] Zhang Z. Yuan B. Bao M. Lu N. Kim T. Liu Y. J. (2011b). The helicase DDX41 senses intracellular DNA mediated by the adaptor STING in dendritic cells. Nat. Immunol. 12, 959–965. 10.1038/ni.2091 21892174 PMC3671854

[B40] Zhao T. Yang L. Sun Q. Arguello M. Ballard D. W. Hiscott J.. (2007). The NEMO adaptor bridges the nuclear factor-kappaB and interferon regulatory factor signaling pathways. Nat. Immunol. 8, 592–600. 10.1038/ni1465 17468758

[B41] Zhou X. Luo J. Mills L. Wu S. Pan T. Geng G.. (2013). DDX5 facilitates HIV-1 replication as a cellular co-factor of Rev. PLoS ONE 8:e65040. 10.1371/journal.pone.0065040 23741449 PMC3669200

