# Supplementary material for: DEAD-Box Helicase DDX25 Is a Negative Regulator of Type I Interferon Pathway and Facilitates RNA Virus Infection
Source: Front Cell Infect Microbiol. 2017 Aug 4;7:356. doi: 10.3389/fcimb.2017.00356 (PMC5543031; doi:10.3389/fcimb.2017.00356)
Supplement: Supplementary file 1 [file Table1.DOC]

Table S1. Oligo-primer sequences for SYBR ® qRT-PCR Assay.

| Target sequence（5’→3’） |
| --- |
| *DDX25* 5’-AACGCTAAGTGGTTGACCGT-3’  5’-TTCACATCAATCCCTCGGGC-3’  *Ddx25* 5’-AGCAATGCTAAGCCGAGTGA-3’  5’-TGCCCATCCTTTCAACCACA-3’  *Human beta-actin* 5’-*GGGCATGGAGTCCTGTGGCA*-3’  5’-*GGGTGCCAGGGCAGTGATCTC*-3’  *Mouse beta-actin* 5’-ACACAGTGC TGTCTGGTGGT-3’  5’-CTGGA AGGTG GACAGTGAGG-3’  *IFNβ* 5’-CATTACCTGAAGGCCAAGGA-3’  5’-CAGCATCTGCTGGTTGAAGA-3’  *Ifnβ*  5’-ACTGCCTTTGCCATCCAAGA-3’  5’-AGTTGAGGACATCTCCCACG-3’  *Ifnα* 5’-GGATGTGACCTGCCTCAGAC-3’  5’-GCTGGGTCAGCTCTTGTAGG-3’  *Ifnγ* 5’-CGGCACAGTCATTGAAAGCC-3’  5’-TGCATCCTTTTTCGCCTTGC-3’  *Tnfα*  5’-GATTCCCCCAGGAGAAGGTG-3’  5’-CATCAGACAGCCTTGCAGGT-3’  *Cxcl1* 5’-*ACTCAAGAATGGTCGCGAGG*-3’  5’-ATAGTGTTGTCAGAAGCCAGC-3’  *Cxcl2* 5’-GGCGGTCAAAAAGTTTGCCT-3’  5’-TTCTTCCGTTGAGGGACAGC-3’  *Ccl5* 5’-AGCAGCAAGTGCTCCAATCT-3’  5’-AGAGCAAGCAATGACAGGGA-3’  *DENV E* 5’-CATTCCAAGTGAGAATCTCTTTGTCA-3’  5’-CAGATCTCTGATGAATAACCAACG-3’  *VSV G* 5’-ACGGCGTACTTCCAGATGG-3’  5’-CTCGGTTCAAGATCCAGGT-3’  *ZIKV E* 5’-GCTGCCCAACACAAGGTGAA-3’  5’-ATGTCACCAGGCTCCCTTTG-3’ |
